# Ch’ol nomenclature for soil classification in the ejido Oxolotán, Tacotalpa, Tabasco, México

**DOI:** 10.1186/s13002-018-0236-5

**Published:** 2018-05-30

**Authors:** Rufo Sánchez-Hernández, Lucero Méndez-De la Cruz, David J. Palma-López, Francisco Bautista-Zuñiga

**Affiliations:** 1 División de Ciencias Agropecuarias, Universidad Juárez Autónoma de Tabasco, Carretera Villahermosa-Teapa km. 25, Ranchería La huasteca, 86280 Centro, Tabasco Mexico; 2 Colegio de Postgraduados, Campus Tabasco, 86500 Cárdenas, Tabasco Mexico; 3 Centro de Investigaciones en Geografía Ambiental, Universidad Nacional Autónoma de México, Antigua Carretera a Pátzcuaro núm. 8701, col. Ex-hacienda de San José de la Huerta, C. P. 58190 Morelia, Michoacán Mexico

## Abstract

**Background:**

The traditional ecological knowledge of land of the Ch’ol originary people from southeast Mexico forms part of their cultural identity; it is local and holistic and implies an integrated physical and spiritual worldview that contributes to improve their living conditions. We analyzed the nomenclature for soil classification used in the Mexican state of Tabasco by the Ch’ol farmers with the objective of contributing to the knowledge of the Maya soil classification.

**Methods:**

A map of the study area was generated from the digital database of parcels in the ejido Oxolotán in the municipality of Tacotalpa, to which a geopedological map was overlaid in order to obtain modeled topographic profiles (Zavala-Cruz et al., Ecosistemas y Recursos Agropecuarios 3:161–171, 2016). In each modeled profile, a soil profile was made and classified according to IUSS Working Group WRB (181, 2014) in order to generate a map of soil groups, which was used to survey the study area with the participation of 245 local Ch’ol farmers for establishing an ethnopedological soil classification (Ortiz et al.: 62, 1990). In addition, we organized a participatory workshop with 35 people to know details of the names of the soils and their indicators of fertility and workability, from which we selected 15 participants for field trips and description of soil profiles.

**Results:**

The color, texture, and stoniness are attributes important in the Ch’ol nomenclature, although the names do not completely reflect the visible characteristic of the soil surface. On the other hand, the mere presence of stones is sufficient to name a land class, while according to IUSS Working Group WRB (181, 2014), a certain amount and distribution of stones in the soil profiles is necessary to be taken into consideration in the name. Perception of soil quality by local farmers considers the compaction or hardness of the cultivable soil layer, because of which black or sandy soils are perceived as better for cultivation of banana, or as secondary vegetation in fallow. Red, yellow, or brown soils are seen as of less quality and are only used for establishing grasslands, while maize is cultivated in all soil classes.

**Conclusions:**

Farmers provided the Ch’ol nomenclature, perceived problems, and uses of each class of soil. Translation of Ch’ol soil names and comparison with descriptions of soil profiles revealed that the Ch’ol soil nomenclature takes into account the soil profile, given it is based on characteristics of both surface and subsurface horizons including color of soil matrix and mottles, stoniness, texture, and vegetation.

## Background

In recent years and in different parts of the world, the importance of local knowledge has been reassessed for the evaluation of natural resources such as soil [1–3], plants [4], animals [5], and ecosystems [6].

During the past four decades, local knowledge of soil has been revalued, yet it has not always been properly understood [7, 8]. Criticism of ethnopedological studies has been based on three premises: (a) farmers only consider the properties of the superficial layer [1, 2], (b) local soil nomenclature is difficult to apply in soil classification systems [2, 8, 9], and (c) the value of indigenous knowledge is limited to the area in which it developed [1, 2].

These premises have already been invalidated by compelling evidence, [10] reported the existence of the Maya term *Kan kab Lu’um*—meaning “soil yellow under” alluding to the subsurface horizon—and elaborated a hierarchic scheme of Maya soil classification. Evidence is still needed to apply ethnopedological knowledge to the study of extensive territories, as did [11], who applied the soil Maya nomenclature throughout the Mexican state of Yucatan.

However, there is agreement about the practical utility of traditional, local, indigenous, and peasant knowledge for the promotion of agricultural, forestry, and livestock development, because it is an essential communication channel between technicians and local farmers [1, 2, 10, 12].

Traditional ecological knowledge is the product of a cumulative and dynamic process of experiences, which unlike scientific knowledge, is local and holistic and implies an integrated physical and spiritual worldview. Such knowledge is part of the cultural identity of indigenous societies and contributes to improve their life conditions [1, 2, 9–13].

The Ch’ol is a Maya ethnic group originary of the state of Chiapas that emigrated to Tabasco in the 1960s, making their case to be particular. Their communities have to adapt to a new environment by adding to their ecological and agricultural tradition the newly acquired adaptive knowledge.

The objective of this study was to analyze and record the knowledge of soils of the Ch’ol farmers in order to contribute to the Maya soil classification.

## Methods

### Description of the study area

The Ch’ol from the community of the ejido Oxolotán—within the municipality of Tacotalpa in the state of Tabasco, Mexico—belong to an ethnic group of Maya origin that was initially settled in the vicinity of the Lacandon jungle in the state of Chiapas. In the decade of the 1960s, a group of Ch’ol people moved to Oxolotán due to its proximity and to the similarity of the climate and vegetation relative to those in their place of origin. In the Ch’ol culture, the elders are respected for their wisdom about cultivation of the milpa. Their primary activity is agriculture, focused on the cultivation of maize, banana, cocoa, and vegetables and the raising of cows in small areas of grasslands and of pigs and chickens in backyards [14].

The ejido Oxolotán is located within the Sierras de Chiapas and Guatemala physiographic province. Geomorphologically, the landscapes are formed by intermontane valleys, hills, and mountains with elevations of 40 to 1020 m a.s.l. and slopes of between 6 and 100%. Soil parent materials can be clastic detrital rocks, limestones, sandstones, limonites, shales, and conglomerates [15]. The climate is Af(m), defined as warm humid with year round rains. Vegetation is composed of the last remnants of the Tabasco rain forest [16].

### Soil survey

The National Agrarian Register (*Registro Agrario Nacional*) provided the digital database of parcels forming the ejido Oxolotán. The resulting map of parcels was overlaid to the geopedological map.

A communitarian assembly was held to present the project, and 245 farmers were invited to participate voluntarily in a community workshop in which spontaneously, and in an open dialog, they were inquired about the knowledge of their lands and more specifically about their identification, nomenclature, use, and management [10, 17]. A total of 35 people participated in the workshop, but the largest amount of information was provided by 15 selected participants who were permanent residents of the community, were dedicated to agricultural activities, and spoke the Ch’ol language.

In the field, four sites of each soil group—which were previously selected on the previously elaborated geopedological map—were visited with individual participants. On the ground, the following questions were asked to participants: How is this soil called? Why do you call it that? How is this soil? How do you distinguish one soil from another? Is this soil the same as that of the neighboring plots? How do you distinguish the boundaries between soils? How many types of soils are there in the region? What plants do you cultivate in this soil? Which is the most productive soil? For how long have you been cropping in this soil? How do you recognize that the soil is being lost?

**Fig. 1:**
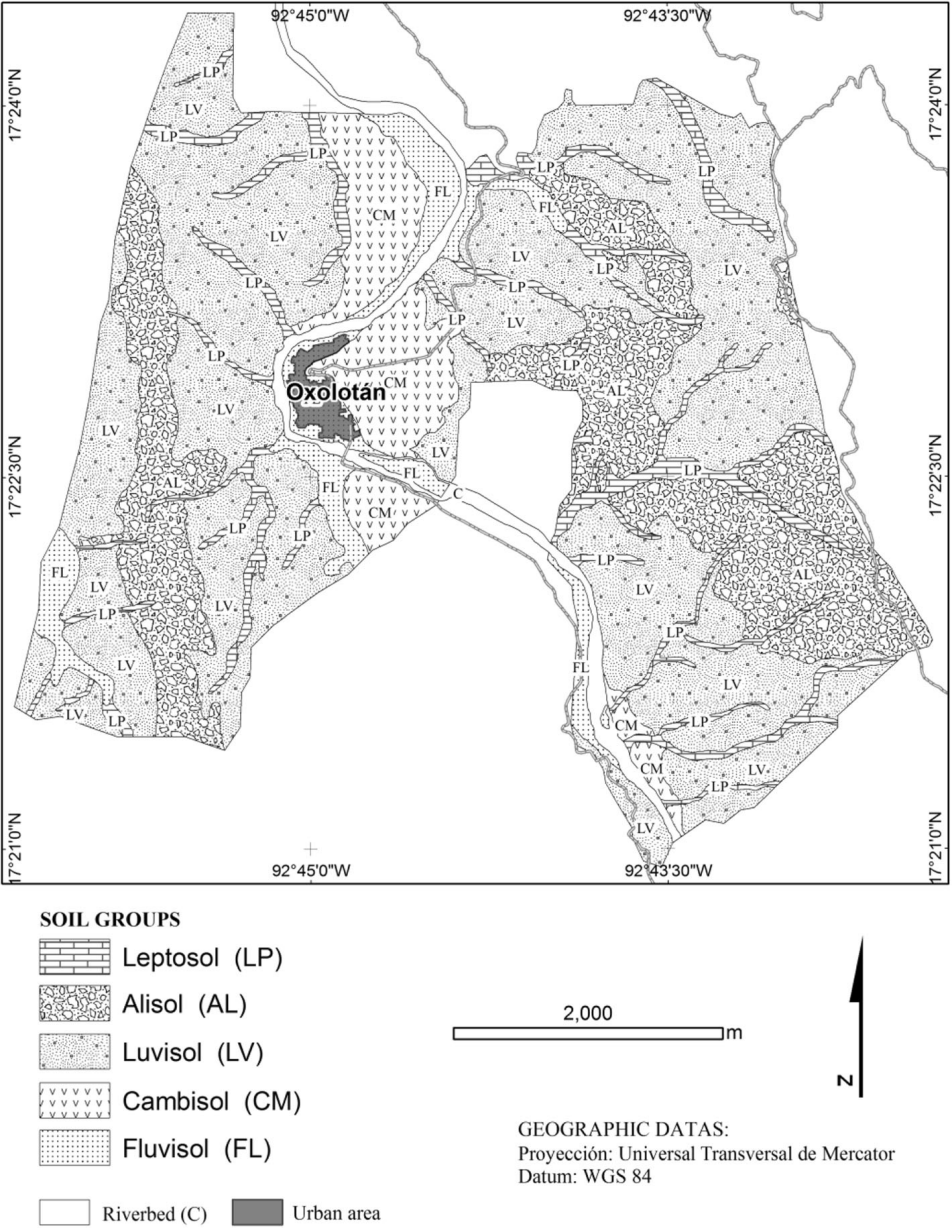
Soil map obtained under the geopedological focus

Prospection of the study area was made in the field with participation of local farmers. Soil profiles were described according to the manual of [18] and samples were taken from each one of the horizons in the soil profiles (Fig. 1).

Soil samples were taken to the laboratory for analysis of pH [19], electrical conductivity (EC) [20], total organic matter content (OM) [21], cation exchange capacity (CEC), exchangeable cations [22], and concentration of phosphorous (P) [23]. Table 1 shows the physical and chemical characterization of the Ch’ol land classes. Based on soil profiles, soils were classified according to the WRB 2014 classification [24].

**Table 1:** Physical and chemical characterization of the Ch’ol land classes

Ch’ol soil class	Ho	pH (H_2_O)	EC (ds m^−1^)	OM (%)	P (mg kg^−1^)	BSP	K	Ca	Mg	Na	CEC	Clay	Silt	Sand
							Cmol^(+)^ kg^−1^					(%)		
*Yiq’uel lum*	Ap	7.56	2.63	3.20	3.92	74.7	0.28	10.48	0.21	0.10	14.81	26	5	69
	C1	8.04	1.70	0.39	3.73	87.3	0.25	9.05	0.66	0.10	11.52	24	2	74
	C2	8.08	1.70	0.46	3.77	90.3	0.24	11.01	0.78	0.10	13.44	26	5	70
	C3	8.04	1.69	0.26	3.98	79.1	0.31	11.32	0.85	0.10	15.91	18	27	56
	2C4	7.87	1.91	0.77	3.76	73.8	0.37	12.44	0.85	0.10	18.65	28	25	48
*Chachac lum/chʌchʌclumil*	A1	5.81	0.97	4.67	3.76	61.5	0.29	10.77	2.34	0.10	21.94	36	33	31
	A2	5.34	0.56	2.26	3.65	49.3	0.22	8.57	1.66	0.10	21.39	38	32	31
	Bw	5.11	0.38	0.49	3.63	36.9	0.31	7.75	1.76	0.10	26.88	40	31	29
	C	5.07	0.29	0.16	3.83	45.8	0.21	6.32	1.66	0.10	18.10	34	28	38
*K’an kab lum*	Ap	5.89	1.87aaå	6.78	3.77	40.0	0.40	13.09	2.31	0.10	39.79	34	31	35
	Bt1	6.07	1.83	4.84	3.70	48.4	0.27	8.53	1.94	0.09	22.38	48	33	19
	Bt2	6.12	1.18	1.98	3.94	60.6	0.26	11.04	1.38	0.09	21.08	60	31	9
*Ji’il lum or ji’lumil*	Ap	5.84	0.90	5.65	4.44	42.0	0.29	8.98	1.94	0.09	26.88	34	31	35
	Bt1	6.19	1.27	3.25	4.69	39.9	0.23	9.62	1.66	0.09	29.07	48	29	23
	Bt2	6.62	1.32	2.02	4.60	59.5	0.27	18.88	1.64	0.09	35.10	54	21	25
*Lum ambʌ ti xajlelol*	Ap	5.54	0.78	4.91	4.61	52.9	0.50	5.98	0.98	0.09	14.26	28	33	39
	A2	5.22	0.58	2.35	4.64	49.9	0.35	5.60	1.07	0.09	14.26	33	34	33
	Bt1	5.16	0.27	1.22	4.48	15.8	0.19	1.62	0.88	0.09	17.55	41	30	29
	Bt2	4.94	0.25	0.20	4.61	20.9	0.18	2.81	1.03	0.10	19.75	41	22	37
*Chac lum or Chʌc lum*	A1	7.43	2.32	1.50	4.33	19.3	0.24	6.31	1.04	0.10	39.79	17	20	63
	C1	7.85	1.88	0.72	4.68	45.2	0.20	8.80	1.03	0.09	22.38	15	20	65
	C2	7.74	2.08	1.43	4.56	43.2	0.19	7.81	1.01	0.09	21.08	17	20	63

P, Ca, Mg, and Na correspond to the chemical element symbols

*Abbreviations*: *Ho* horizon, *EC* electric conductivity, *OM* organic matter, *CEC* cation exchange capacity, *pH* hydrogen potential

## Results

### Description and classification of soils

The Ch’ol of the community of Oxolotán use two types of categories to classify and name their soils. Some categories are qualitative and can be perceived by the senses, such as color, texture, stoniness, and color of the topsoil or the underlying layer. Other categories are based on the capacity for agricultural use of land and plant cover. In the best classes of soil any crop can be developed, while in the regular or bad soils, only milpa can be cultivated. The Ch’ol soil classification considers the superficial compaction (hard or soft) and the problems soils present for management (erosion and land depletion). With a sample of 10% of the Ch’ol population of the Oxolotán community, there was a general consensus of around 90% on the criteria for classifying and naming their soils. Ninety-one percent of the interviewees were capable to identify and name the best and worst soils, the crops that could be established in each one, and the problems presented by each soil class.

The six classes of soils were identified and named by local farmers according to their attributes of surface color, texture, stoniness, and color of the subsurface horizon, as described below (Table 2).

**Table 2:** Names and characteristics of the Ch’ol land classes and their equivalent technical names

Ch’ol name	Description	WRB soil group	Fertility/workability	Crops	Problems for agricultural use
*Yiq’uel lum*	Black land fertile of the riverside	Calcaric Fluvisols (Loamic)	Good/soft	Home garden/acahual^a^	Insufficient fallow period
*Chachac lum/chʌchʌclumil*	Reddish soil in the C horizon	Leptic Chromic Dystric Cambisol (Loamic)		Home garden/pasture/milpa^b^/acahual	Erosion
*K’an kab lum*	Yellow soil in B horizon	Leptic Luvisol (Clayic, cutanic, epidystric, humic)	Regular/regular		Erosion
*Ji’il lum or ji’lumil*	Sandy soil	Leptic Calcaric Fluvisol (Loamic)	Good/soft	Milpa and banana cultivation	Insufficient fallow period
*Lum ambʌ ti xajlelol*	Stony soil or without vegetation cover	Hyperskeletic Leptosol/Skeletic Leptic Luvisol Clayic, Cutanic, Epidystric	Bad/hard	Milpa	Erosion
*Chac lum or Chʌc lum*	Red land (with red mottles)	Leptic Rhodic Alisol (Cutanic)	Regular/regular	Milpa/home garden	Erosion

Notes: ^a^Nahuatl term for naming an abandoned milpa (land that a few years ago was milpa and is now covered by trees)

^b^Maya term for naming the traditional management of maize cultivation

#### *Yiq’uel lum* (fertile fluvial black soil)

The Ch’ol term *yiq’uel* refers to river and the term *lum* means soil; however, when speaking Spanish, local farmers call these soils *tierra negra*, meaning black soil, because of which it may be proper to refer to these soils as fertile fluvial black soils. Ch’ol farmers perceive that manageability of these soils is intermediate, meaning their hardness or compactness is intermediate (Table 2). The participants referred to these soils as suitable for growing maize, bananas, *yucca* (manioth, *Manioth esculenta*), and forest trees such as *cedro* (Spanish cedar, *Cedrela odorata*). Other uses of these soils are for home gardens and for secondary vegetation in fallow (in Spanish, *acahuales* or *monte*).

#### *Ji’il lum* or *ji’lumil* (sandy soils)

The Ch’ol names *ji’il lum* and *ji’lumil* mean sand or sandy soil. Local farmers think these soils are suitable for growing maize and bananas and that their quality is regular or bad, because of which they are generally used for grasslands, milpa, and secondary vegetation. Ch’ol farmers said that the main problems of these soils are the landslides; a phenomenon known locally as *yejmel*, this term is translated to Spanish as *derrumbe*.

**Fig. 2:**
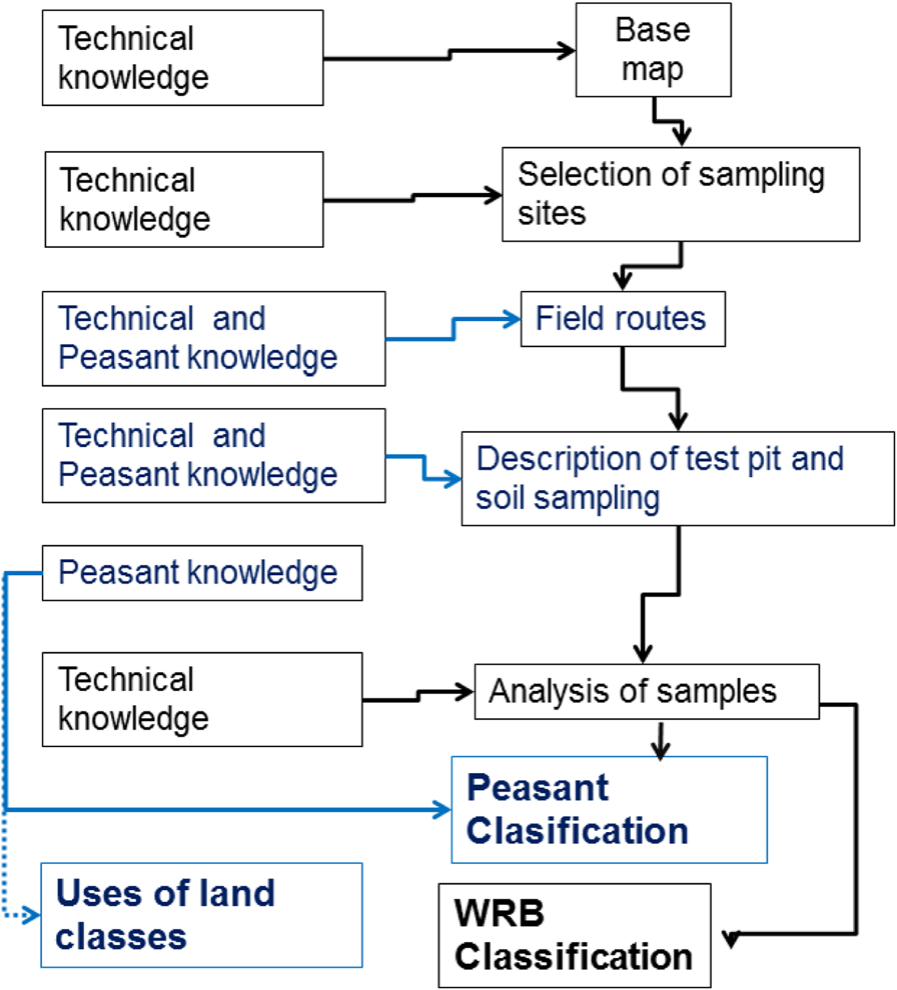
Methodological diagram

These soils (*ji’il lum or ji’lumil*) develop in alluvial islands and in river margins (Fig. 2) and have only a shallow A horizon (15 cm deep) over a C horizon going from 15 to 70 cm in depth, under which there is parent material formed by rocks and rounded pebbles evidencing fluvial processes during soil formation. The color of the surface is brown (10YR3/2), and the C horizon is yellowish brown (10YR4/2) above and in the deeper part it turns brown (10YR3/3) due to eluvium and illuvium of organic matter. The texture of these soils makes them highly permeable, and their structure is moderately developed in the surface, but weakly developed in deeper horizons. Their consistency is friable, sticky, and slightly plastic.

According to technical description, these soils correspond to fertile fluvial black soils and have a brown (10 YR4/3) Ap horizon over a yellowish brown (10YR5/6) C horizon. Their structure is well-developed polyhedral with fine to medium subangular blocks.

#### *Chachac lum* or *chʌchʌclumil* (soil red under)

The Ch’ol term *chachac lum* or *chʌchʌclumil* means red soil, but refers to the color of the subsoil, given that the color of the soil surface is brown. These soils are used for cultivating cocoa trees, bananas, and forest trees and are considered by farmers as of good quality, but mentioned that their management is difficult because of their hardness. Red soils develop on hills, plateaus, and hill summits. According to local farmers, the major problem associated to these soils is their erosion.

The profile of these soils presents a 35-cm-deep A horizon of dark yellowish brown (10YR4/4) color that is slightly lighter (10YR5/4) in the upper 10 cm. Between 35 and 60 cm in depth, there is a Bt horizon with characteristics similar to those of the A horizon; between 60 and 87 cm in depth, there is a light red (5 YR 6/8) C horizon; and from 87 cm deep, there is continuous weathered rock. Along the profile, the structure is strongly developed in fine to medium subangular blocks and their texture is clayey loam. Porosity is frequent throughout the profile, becoming slightly more abundant in the A horizon. Medium and fine roots are frequent and there is presence of earthworms.

#### *K’an kab lum* (soil yellow under)

The meaning of the Ch’ol name of this soil class is “yellow under,” which makes reference to the color of the B horizon. In the study area, the more common use of yellow under soils is for milpa, grassland, and secondary vegetation, although home gardens are also present in it. Their main risk of degradation is from erosion. These soils are located in plains and small hills.

Drainage is good throughout the profile. The surface presents a dark brown (10YR2/2) A horizon reaching 35 cm in depth with few stones. In depths above 35 cm, there is an irregular B horizon with accumulation of brown (10YR3/2) and alternating yellowish brown (10 YR 4/4) clay illuvium. The A and B horizons are underlain by parent material of limonite and sandstone at a depth of less than 90 cm. Along the profile, there is strongly developed structure of fine, medium, and large polyhedrons and subangular blocks, and the texture is of clayey loam giving these soils a friable, sticky, and plastic consistence.

#### *Lum ambʌ ti xajlelol* (rocky or bare soil)

The Ch’ol name *lum ambʌ ti xajlelol* means soil without herbaceous plant cover. For Ch’ol farmers, these soils are hard and difficult to work, and their main causes of degradation are erosion and landslides. They are generally used as cropland, mainly for growing maize. These lands are unsuitable for cultivation due to compaction and stoniness. However, the Ch’ol value these soils because they can at least grow maize on them, besides that the largest area of the Oxolotán lands corresponds to this soil class.

The stony or bare soil areas include two different soil types that are an association of Hyperskeletic Leptosol and Leptic Luvisol (Clayic, cutanic, epidystric, humic). Due to the high number of rock croppings in these soils, they are mainly identified as having large amount of rocks and scarce amounts of fine soil, characteristics that perfectly match with their designation as rocky soils. These soils distribute on convex hill slopes, and in the presence of erosion, they display rock croppings.

In places where fine soil is more abundant, soil is shallow, although they have a considerable development, which is evidenced by presence of a thin, approximately 15-cm-thick A horizon containing over 5.5% of organic matter, which gives it a dark brown (10YR2/2) color that becomes lighter with depth. The color of the B horizon goes from brown (10 YR 3/2) to yellowish brown (10YR5/4). In the surface, the texture is clayey loam and becomes more clayey at increasing depths. These soils generate a strongly developed structure along the profile, mainly in polyhedrons and subangular blocks, and a sticky and plastic consistency.

#### *Chac lum* (soil with red mottles)

The Ch’ol name *chac lum* is literally translated as red soil, but it refers to the red mottles present in the soil matrix. These soils are hard and are difficult to work with, because of which they are used for grasslands and maize cultivation. The main restriction for agriculture of these soils is their risk of erosion, because of which local farmers categorize this soil class as of regular quality. According to the Ch’ol, these lands are easy to distinguish and they know where they are distributed, besides that in these places the grass does not grow because the land is fatigued (their fertility has been depleted). Therefore, it is necessary to let them rest during the dry season.

Red mottled soils develop on convex hill slopes, because of which they have adequate drainage with donor character. The A horizon is approximately 20 cm deep and is underlain by a B horizon between 20 and 60 cm in depth. Below the B horizon, there is a layer of cemented, impermeable material going to a depth of 100 cm. The surface is brown (10YR3/2) with common fine, subtle dark red (7.5YR3/2)-colored mottles due to a combination of organic matter and oxidized clays, colors that in the B horizon become yellowish to yellow brown (10 YR 5/4 to 10 YR 6/6).

## Discussion

The consensus level in the answers on the ethnic knowledge of the soil nomenclature and land use was 91%; only 9% of the interviewees could not give a precise term to the different land classes that were presented during the workshop and the field tour. To the question of why the inhabitants considered that some people were unable to give a correct name to the land classes, 85% of the interviewees attribute it to the fact that the Ch’ol language has many variants, since there is a Ch’ol language that arrived with the people originated from Tila, Chiapas, who came to Oxolotán in the middle of the past century, while there are other people who were already in the place and that dominated the Zoque language—a language of different origin from the Maya—so there is currently a mixture between both dialects [25]. Another factor of heterogeneity of the information is the age and activity of the people, according to the interviewees, the youngest people of the community—not totally dedicated to the agricultural activity—do not speak the Ch’ol language correctly or can fall into inaccuracies when they offer information.

The *yiq’uel lum* and *ji’il lum* or *ji’lumil* soils correspond to Fluvisols [15]. In the case of the ejido Oxolotán, although its formation is conditioned to flat reliefs located between hills, it allows the organic or inorganic sediments to accumulate as in valleys, marginal islands, or even riverbanks. In this sense, the ethnic knowledge had very present the importance played by the relief in soil nomenclature, which allowed 100% of Ch’ol farmers to distinguish clearly between *yiq’uel lum* and *ji’il lum* or *ji’lumil* across the proximity to a river, and although the term *yiq’uel lum* refers to the black color of the land, in the worldview of the Ch’ol, it is clear that this color is due to the presence of the river that enriches the lands.

Other criterion utilized by the Ch’ol is soil color, some examples of that are the cases of the *chachac lum* or *chʌchʌclumil*, *k’an kab lum* and *chac lum* soil classes. However, the difference between these soil classes is perceptible to 91% of the Ch’ol farmers, since the *chachac lum* or *chʌchʌclumil* and *k’an kab lum* soils have a perceptible color contrast between surface and sub surface horizons, while the *chac lum* soils lack that contrast. Both the Maya and the Ch’ol use this same criterion to classify and name soils, as well as to recognize the limitations that these have for cultivation of milpa, so the supply of manures, manual tillage, and use of cover crops are common practices that the Maya use to conserve their soils [10], something that is not observed among the Ch’ol community.

*Yiq’uel lum* is classified as Leptic Calcaric Fluvisol [24], having a primary qualifier Leptic (le) means that they continuous rock ≤ 100 cm from the soil surface, in addition to presenting a Calcaric (ca) supplementary qualifier.

The *yiq’uel lum* soils are similar to the *ji’il lum* or *ji’lumil* soils; however, the Ch’ol indicate that in addition to the superficial dark color, another way of identifying these soils is their proximity to the river bank, the *yiq’uel lum* being further away from the river bed and capable of developing any crop, while *ji’il lum* or *ji’lumil* soils are at the river bank and only banana is grown. However, although both soils are similar for the Ch’ol and the names do not reflect a single specific characteristic observable in each soil class, they imply other important aspects such as their location in the landscape as well as their capacity for agricultural use, as is the case of the Mayan soil names in the state of Yucatan [10, 11, 26].

*Yiq’uel lum* and *ji’il lum* or *ji’lumil* are two soil classes that have the same technical name (Leptic Calcaric Fluvisol) [24] according to the international nomenclature system, but for the Ch’ol farmers, the difference between the two classes is their location in the landscape.

The Ch’ol nomenclature considers attributes that are not visible on the soil surface. The classes *chachac lum* or *chʌchʌclumil* and *k’an kab lum* correspond to Chromic Dystric Cambisol (Loamic) and Leptic Luvisol Clayic (Cutanic, epidystric, humic), respectively, according to the classification of [24]. Both names refer to the clay content, which requires mechanical analysis for its determination, but the ethnic communities do not limit their observation to the soil surface, an example being the term *k’an kab lu’um* which is used by the Maya to indicate the presence of a yellow horizon below the surface [10, 27].

The terms *chachac lum* or *chʌchʌclumil* and *k’an kab lum* refer to the color of the subsoil. For the first time, it is reported that the name of a soil (*chachac lum* or *chʌchʌclumil*) is due to the color of the mottles instead of the color of the soil matrix. The Ch’ol indicate that both soils are similar, although the *chachac lum* soils are softer and deeper than the *k’an kab lum* soils.

**Fig. 3:**
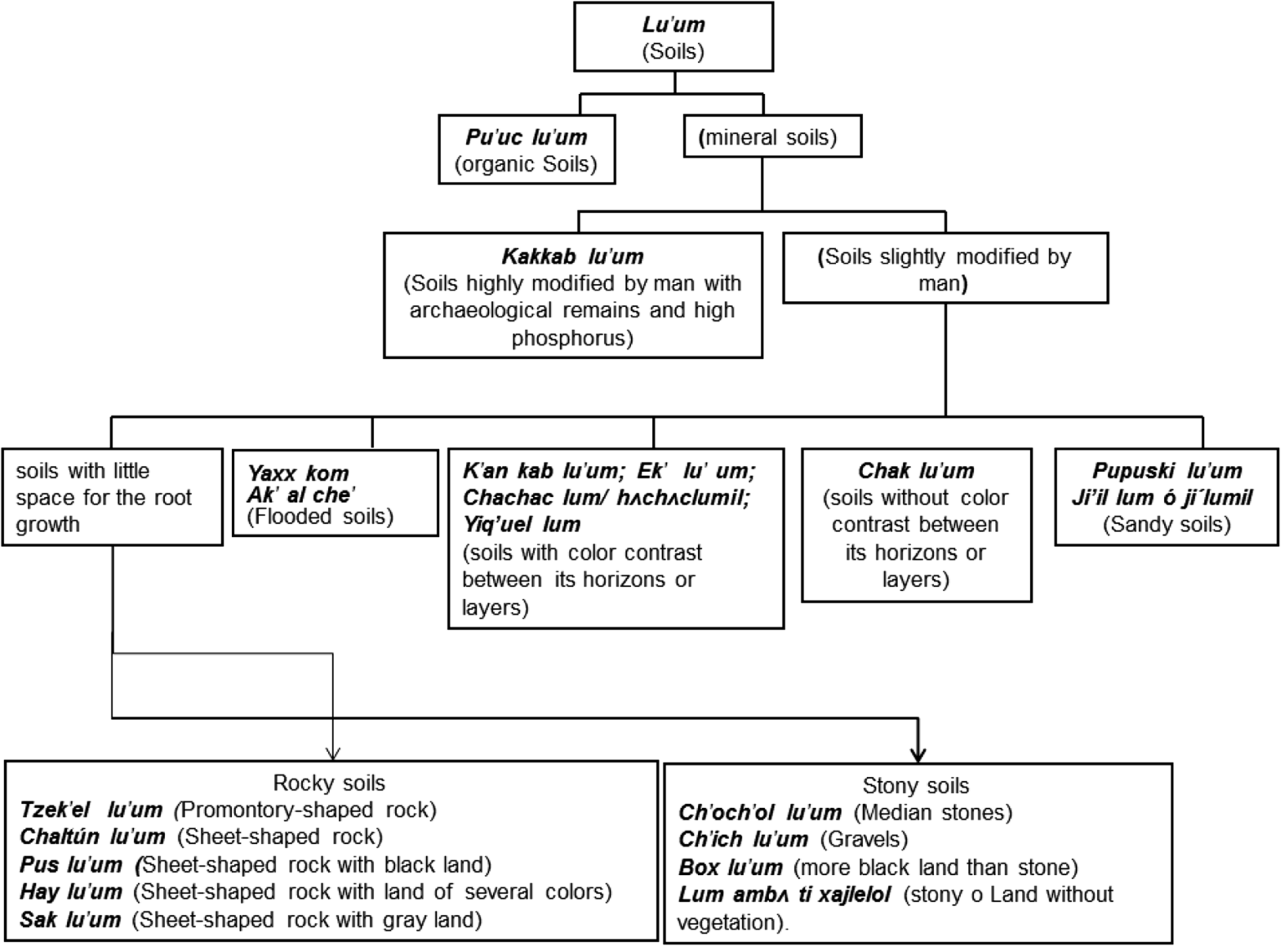
Mayan soil classification and the new contributions of the Ch’ol soil nomenclature

This Ch’ol soil name is similar to *chac lu’um* of the Maya soil classification, but the terms are not equivalent rather being synonyms [7, 10, 27] (Fig. 3).

The Ch’ol nomenclature of the soil classes is similar to other ethnic nomenclatures, particularly in the breadth of the terminology, since the farmers recognize soils with an accuracy of more than 90% in the presence of rocks and other characteristics visible in the landscape, for example, low vegetation or the difficulty of work.

The original names of soil classes indicate the characteristics or properties that identify it, such as the color of the matrix and mottles, texture, stoniness, rockiness, consistency, retention of humidity, tillage, and fertility, among others [28]. In addition, farmers use the names of the soil classes to locate them geographically [8].

The stoniness and rockiness are attributes utilized as classification criterion, both in the technical [24] and in the ethnic [10, 11, 26, 29–33] classifications (Table 3). The difference is that in the indigenous classifications such as the Maya, the stoniness and rockiness is a property that determines the name of the soil (Fig. 3), while in technical soil classifications, this is not the case with the exception of the Leptosol group in WRB classification [24].

**Table 3:** Local criteria for soil classification used by several ethnic groups of Mexico

Criteria	Ch’ol	Maya	Purépecha	Nahuatl/Otomí	Mixe
Climate and altitude at the local or regional level.	Landscape	Landscape	Climate/landscape	Landscape	Landscape
Organoleptic properties.	Color of the horizons (matrix and mottles), stoniness, rockiness, moisture	Color of the horizons, stoniness (size and abundance) gravel content, rockiness (shape and abundance), moisture, depth	Color of the horizons, stoniness	Soil color, stoniness, moisture	Soil color, stoniness, moisture
Quality/fertility	Texture, compaction, salinity	Organic matter, texture	Organic matter, texture	Texture plasticity compaction	Texture, plasticity, salinity and compaction
Productive capacity	Good soils Bad soils	All soils have uses	Good soils Currents	Good soils Bad soils	Good soils Bad soils
Workability (Consistence)	Loose/hard		Loose/Soft	Loos/hard	Loose/hard
Land use/coverage	With or without vegetation	Hydrophilic plants/seasonal herbs/specific trees for each soil and other soils without vegetation	Forest land/crop land		
References	Own elaboration	[10, 30–33]	[29]	[12, 34]	[3]

Both the Maya and the Ch’ol soil classifications have names for sandy soil, such as *pupuski lu’um* and *ji’il lum*, the former referring to Arenosol [10, 27] and the latter to Fluvisol. The Ch’ol term *lum* is nearly equivalent to the Maya term *lu’um*, both meaning soil. Ch’ol farmers classify the quality of soil by means of properties such as the degree of superficial hardness or workability, ranging it from regular to bad. According to [34], the farmer’s conception of quality of soil types corresponds not only to the soil’s attributes but also to the identification of advantages and limitations of the environment and the soil (i.e., fertility, humidity, drainage), which translates into technology adapted to specific conditions. As found by [3] in Oaxaca, the nomenclature given by Ch’ol farmers to soil classes is based on their perception of humidity retention, size and content of clasts, and color of mottles.

## Conclusions

Precise translation of names given by Ch’ol farmers to soil types and their comparison with technical descriptions of soil profiles revealed that they take into account subsurface horizons, soil matrix and mottle colors, clasts, texture, and presence of vegetation.

Our study found two cases of inclusion of subsurface horizons in Ch’ol soil nomenclature: (a) the term *kan kab lum* meaning yellow soil below in reference to horizon B and (b) *chachac lum* or *chʌchʌclumil* in reference to the red color of mottles in the C horizon.

The Ch’ol farmer’s perception of soil quality is primarily associated with the degree of compaction or hardness of the cultivable soil layer, because of which black or sandy soil is considered favorable, but red soil is considered undesirable due to its superficial compaction. Maize cultivation is distributed in all classes of soil, while grasslands are restricted to minor quality soils, such as red, yellow, or brown and red, and the best quality soils (black and sandy) are considered to be suitable for banana cultivation.

## Abbreviations

Af(m): Warm humid with year round rains
CEC: Cation exchange capacity
EC: Electrical conductivity
OM: Organic matter
pH: Hydrogen potential
PSB: Base saturation percent
WRB: World Reference Base
